# “I didn’t even know what I was looking for”: A qualitative study of the decision-making processes of Canadian medical tourists

**DOI:** 10.1186/1744-8603-8-23

**Published:** 2012-07-07

**Authors:** Rory Johnston, Valorie A Crooks, Jeremy Snyder

**Affiliations:** 1 Department of Geography, Simon Fraser University, 8888 University Drive, Burnaby, BC, Canada; 2 Faculty of Health Sciences, Simon Fraser University, 8888 University Drive, Burnaby, BC, Canada

**Keywords:** Medical tourism, Decision-making, Qualitative, Surgery, Canada

## Abstract

**Background:**

Medical tourism describes the private purchase and arrangement of medical care by patients across international borders. Increasing numbers of medical facilities in countries around the world are marketing their services to a receptive audience of international patients, a phenomenon that has largely been made possible by the growth of the Internet. The growth of the medical tourism industry has raised numerous concerns around patient safety and global health equity. In spite of these concerns, there is a lack of empirical research amongst medical tourism stakeholders. One such gap is a lack of engagement with medical tourists themselves, where there is currently little known about how medical tourists decide to access care abroad. We address this gap through examining aspects of Canadian medical tourists’ decision-making processes.

**Methods:**

Semi-structured phone interviews were administered to 32 Canadians who had gone abroad as medical tourists. Interviews touched on motivations, assessment of risks, information seeking processes, and experiences at home and abroad. A thematic analysis of the interview transcripts followed.

**Results:**

Three overarching themes emerged from the interviews: (1) information sources consulted; (2) motivations, considerations, and timing; and (3) personal and professional supports drawn upon. Patient testimonials and word of mouth connections amongst former medical tourists were accessed and relied upon more readily than the advice of family physicians. Neutral, third-party information sources were limited, which resulted in participants also relying on medical tourism facilitators and industry websites.

**Conclusions:**

While Canadian medical tourists are often thought to be motivated by wait times for surgery, cost and availability of procedures were common primary and secondary motivations for participants, demonstrating that motivations are layered and dynamic. The findings of this analysis offer a number of important factors that should be considered in the development of informational interventions targeting medical tourists. It is likely that trends observed amongst Canadian medical tourists apply to those from other nations due to the key role the transnational medium of the Internet plays in facilitating patients’ private international medical travel.

## Background

The term ‘medical tourism’ describes the intentional movement of patients across international borders to seek medical care that has been privately purchased and arranged for [1,2]. The elements of intentionality and private arrangement are key to defining which care-seeking behaviours constitute medical tourism as opposed to other forms of international medical travel such as formal cross-border care arrangements and emergency care for vacationing tourists, although the term has been used at times to describe all of these forms of care. The global medical tourism industry is steadily growing, although accurate estimates of its current size or scale are not available given the presence of exaggerated figures and inconsistencies in tracking flow numbers, in part due to a poor universal definition of what constitutes medical tourism [3,4]. Despite this, it is known that steady flows of patients traveling from the Global North (e.g., Canada, the United States [US], Western Europe, Australia) to clinics in the Global South (e.g., India, Thailand, Costa Rica) have emerged over the past decade [5,6]. These new patterns of trade have joined the long-established South–north and North-North flows of international patients to internationally reputed medical centres, such as the Mayo Clinic in the US, as well as existing flows of patients between Southern nations [7,8]. The growth of the medical tourism industry has been made possible by increasingly globalized flows of trade, transportation, and information [5,9]. In turn, medical tourism ties the interests of disparate populations together, for example by introducing novel global pathways for the spread of infectious disease and through the sharing of scarce health resources amongst citizens of different nations [10,11].

A series of recent scholarly reviews about medical tourism have consistently revealed that there are significant gaps in our understanding of this phenomenon [2,12-14]. In addition, these reviews have indicated that much of the existing knowledge base is derived from speculative claims [3]. These knowledge gaps persist despite an increasing desire amongst global health researchers to better understand medical tourism because of the implications this practice is thought to hold for the equitable delivery of health services, the involvement of new actors (e.g. medical tourism facilitators) in the delivery of health care, and the novel responsibilities of patients seeking and physicians providing health care across international borders, among other concerns [2,15,16]. For example, a scoping review completed by Crooks et al. [12] concluded that we have much to learn about patients’ experiences of medical tourism, including how medical tourists access and evaluate information sources prior to departure. Lunt et al.’s [17] more recent article echoes this conclusion, and identifies patient decision-making as one of the priority areas for medical tourism research given its relevance to continuity of care, patient health and safety, and the commodification of care. While media accounts provide some valuable insights into the experiential dimensions of medical tourism [e.g. [18-20], deep inquiry into the *process* of patients’ medical travel, from conception to return, remains lacking. This absence of knowledge leaves major questions about which factors and actors inform the decision-making of medical tourists, especially in regard to their reliability and modes of dissemination. In this article we address this knowledge gap through examining Canadian medical tourists’ decision-making processes regarding seeking surgery abroad.

Canadians are amongst those participating in the medical tourism industry, not only as patients, but also as investors and facilitators (i.e., agents specialized in coordinating international medical care, including arranging for visas and accommodation and dealing with destination hospitals) [2]. The only quantitative report on medical tourism in Canada produced to-date indicates that 2% of 2,304 Canadian survey respondents have traveled outside the country to “consult with a doctor, undergo a medical test or procedure, or receive treatment” [21]. As a description of how this care has been paid for or arranged is not indicated, other forms of international medical travel (e.g., cross border care arranged through the public system) may be in the estimate. Further, 20% of those surveyed indicated they would travel abroad for private-pay health services [21]. Certainly, this is no reliable indication of how many patients are indeed traveling abroad for private medical care. Numbers aside, it is indeed the case that Canadian patients are choosing to take part in medical tourism, a phenomenon that is receiving increasing media attention in the country [2].

The phenomenon of Canadian patients privately choosing to travel outside of their home health system to access medical care abroad is intriguing, as medically necessary health care in Canada is publicly funded and universal. Federal legislation limits the availability of domestic private health care, making privately purchased, on-demand access for many treatments largely inaccessible to most Canadians [22]. There is no single Canadian health care system, as the management and delivery of health care is the separate responsibility of each of the 13 provinces and territories [23]. Canada’s federal government contributes to the financing of each provincial health system through equalization payments that work to minimize inequities in essential services, with the amount paid to each province differing on the basis of need [22]. The balkanization of the management and financing of the national health system contributes to substantial differences in temporal and spatial access to care across the country for the same procedures or treatments due to differences in the priorities and resources of the health administrations in each province or territory [23]. For example, in 2010 42% of patients in the province of Nova Scotia had timely access to knee replacement surgery (i.e. within the national benchmark period of six months between referral to specialist to surgery), compared with 89% of patients in Ontario [24]. These wait times are likely to serve as a prompt to consider care elsewhere for some Canadian patients [2]. Canadians also travel abroad for non-medically-necessary procedures such as dental care and cosmetic surgeries that are not covered by the public health care system. It has been speculated that procedure costs are likely to serve as motivators for seeking such care abroad for Canadian patients [2].

Much research exists about patients’ decision-making as it pertains to surgical care sought domestically. It has been reported that patients are often hesitant to change surgeons, even if it means an earlier surgical date, suggesting that trust and familiarity with care providers and venues can outweigh other decision-making concerns such as wait times [25,26]. Striking a balance between appropriate preparation times and meeting personal expectations of prompt care is also a factor in patients’ decisions about if and when to receive care. For example, it has been found that Canadian patients appreciate having time to prepare for elective surgery and will seek to organize about two months between the booking date and surgical date into their trajectory of care [27]. However, if this two month threshold is crossed, resentment builds as the wait time is generally perceived to be excessive [28]. Another element of surgical decision-making that has been explored is the sharing of information between physicians and patients. While providing informed consent is a keystone principle of Western clinical practice, it has been reported that the comprehensiveness of information shared between surgeons and patients about the risks and benefits of surgery varies widely [29,30]. The outcomes of this information sharing are thought to influence the willingness of patients to ultimately seek treatment [29]. More generally, it has been shown that the ability of individuals to discern statistical representations of the risks of surgical treatments are greatly influenced by anecdotal accounts of procedure success or failure, suggesting that personal narratives of treatment can be potent influences on patient decision-making [31]. In the current analysis we extend this existing body of knowledge by investigating how the unique logistical and informational challenges posed by privately accessing care internationally as a medical tourist coincide with or depart from receiving surgical care domestically.

Existing understandings of patients’ decision-making for surgical care have yet to consider the unique dimensions of medical tourism, such as concurrently seeking and synthesizing information about surgical treatment, travel, foreign destinations, and how the risks of each may interact to heighten the potential for negative health outcomes [14,32]. While it is often speculated that medical tourists rely primarily on the Internet to inform themselves about destination facilities, the frequency of access to information found online and its actual influence on decision-making requires dedicated attention [33,34]. Furthermore, it is widely reported that medical tourists from particular source countries seek care abroad based on singular motivations found in their home context, such as the high cost of medical care in the US, limited availability of medical care in the Global South, or long wait times for medical care in countries with public health care systems such as Canada [35,36]; yet, this tendency toward simplistic accounts potentially belies complex interaction among the factors that compel individuals to investigate seeking care abroad. We seek to unpack some of these assumptions in the current analysis through examining the experiential accounts of 32 Canadians who sought private surgical care abroad.

The purpose of this article is to shed light onto how Canadian medical tourists go about deciding to access surgery abroad and what kinds of information sources inform their decisions. Our goal is to contribute to developing an empirically-informed knowledge base about the global health services practice of medical tourism through addressing the knowledge gaps identified above. Because of their exposure to a public and universal health care system for medically necessary care at home, Canadian medical tourists encounter an entirely different mode of access to care when privately seeking surgery abroad, foregoing public payment for the ability to determine what kind of care they wish to access, and when [12,37]. Even when seeking surgical care that is not offered through the public system, such as experimental surgeries only available in other countries or cosmetic procedures, Canadian medical tourists are likely to encounter significant differences in facilitating access to medical care abroad than they would domestically. These differences are likely to include protocols around procedure booking and patient record transfer, among other factors [12]. As such, Canadian medical tourists may need to adopt more extensive roles as information assessors and decision-makers than they are used to, shifting them from the more passive role of the traditional patient to the more active, neo-liberalized position of the ‘patient-consumer’ [38].

## Methods

This analysis forms part of a larger exploratory study of the decision-making processes and experiences of Canadian medical tourists. The study involved interviewing Canadian medical tourists and medical tourism facilitators. This analysis exclusively considers the former participant group.

### Recruitment

We sought to recruit Canadians who had previously undergone surgical treatment abroad for semi-structured phone interviews. As there is no organized tracking or surveillance of this patient group, potential participants were identified through numerous decentralized avenues. These included: (1) collecting names of medical tourists from Canadian news reports and contacting them via phone or email; (2) advertising in Canadian print news outlets; (3) posting invitations to participate on online medical tourism forums; (4) snowball sampling through participants’ networks; and (5) providing study details to facilitators to disseminate. No apparent differences emerged between participants based on how they were recruited, such as in their motivations for travel abroad or experiences of medical tourism. People interested in participating in an interview were asked to contact a toll-free phone number or an e-mail address. Detailed study information was provided upon contact and eligibility assessed. Upon establishing a participant’s eligibility, an interview time was then scheduled.

Participation was limited to those who met the eligibility criteria of: (1) having successfully pursued privately-arranged surgery outside of Canada paid for out-of-pocket; (2) being enrolled in a Canadian public health care plan at the time of surgery; and (3) being over the age of 18 at the time of the interview. To maintain focus, participants who went abroad for care other than surgery (e.g., diagnostic testing, tooth cleanings or fillings) were excluded. Those who had procedures that involved third parties (e.g., transplants, some reproductive surgeries) were also excluded. This is because confidentiality cannot be guaranteed to participants who report illegal activities, as per Canadian research ethics policies, and it was thought that there is a risk among this population that such activities would be discussed. Because we wanted to extend confidentiality to participants, we did not include people who had had these surgeries in the study. All those who scheduled an interview followed through with participating and no participants elected to withdraw from the study after being interviewed. Prior to participant recruitment, ethics approval was sought from and granted by the Office of Research Ethics at Simon Fraser University.

### Data collection

Semi-structured interviews were conducted by phone between July and November, 2010. A semi-structured approach was employed to allow common issues to be explored, while giving participants the freedom to introduce unanticipated topics of relevance to their experience. Table 1 includes selected questions from the interview guide. All interviews were conducted by the same investigator (the lead author) in order to enhance consistency. Interviews typically ran for 1–1.5 hours and were digitally recorded. The interviews covered a wide range of topics, including participants’ motivations, assessment of risks, information seeking process, experiences in both the domestic and international health systems, and pursuit of post-operative care. Data collection ceased upon the exhaustion of all of our recruitment methods. This was determined after no new participants were identified through public sources or contacted us after a month-long period.

**Table 1:** Selected Interview Questions

**Selected Questions**	**Sub-Probes**
Tell me about when you traveled to ___________ for surgery.	○ What was it like?○ What procedure did you get?○ How long did you go for?○ Did anyone accompany you?○ Had you been to__________ before?
When was it that you traveled to __________________ for the procedure?	○ For how long before then had you been planning the trip?○ For how long before then did you know or decide that you were going to get that surgery done?
Why did you decide to go to ______________?	○ Did anyone tell you about it?○ What kinds of information did you look at?○ Where did you get this information from?○ Were personal finances an important deciding factor in choosing to go to _________?○ Did you consult with your family doctor about your plan to go abroad for surgery?

### Analysis

All interviews were transcribed verbatim. A review of the transcripts was conducted by all authors. Following initial review, a meeting was held to share impressions of common issues emerging from the interviews. A preliminary coding scheme was constructed, which was structured around the agreed upon issues. Generally speaking, scheme creation involved identifying umbrella terms or concepts to which data segments were assigned that could be drawn together in different combinations and permutations in order to inform a thematic analysis. Coding of the transcripts followed, which was done using NVivo qualitative data management software. To ensure the utility of the codes, one investigator undertook the coding while another reviewed the first coded transcript to confirm the functionality and interpretation of the scheme. Through an iterative process of coding and team discussion, superfluous codes were eliminated and overpopulated single themes were disaggregated as part of a second stage of coding. Upon completion of the coding process, thematic analysis was undertaken. Thematic analysis involves reviewing coded data to finding patterns or trends within the dataset that are compared to study objectives and existing knowledge in order to refine the interpretation of their meaning [39]. By examining full narrative accounts by theme, commonalities in particular domains emerged despite the underlying structural differences (e.g., destination location, procedure type) in the medical tourists’ raw accounts.

## Results

**Figure 1:**
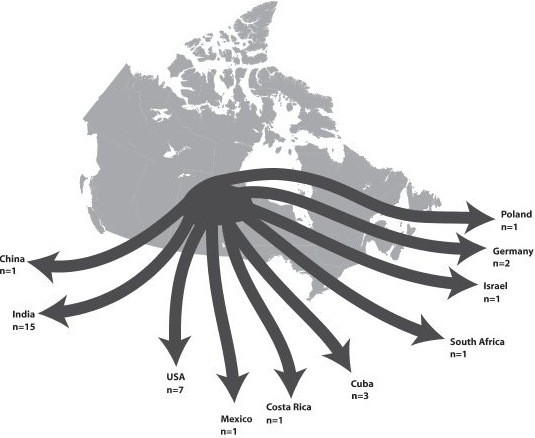
**Destination countries visited by participants.** This figure outlines where participants travelled for their procedures and how many went to each country. Note that one participant travelled to two countries for treatment addressing the same health problem, resulting in a total of 33 unique trips.

In total, 32 medical tourists from eight of Canada’s 13 provinces and territories were interviewed. On average, two years had elapsed from the time of the surgery abroad to the time of the interview, with the longest being six years. Figure 1 and Table 2 provide an overview of some of the participants’ key characteristics. In total, 21 participants sought surgeries that were not available to them in Canada. Of these, six sought procedures not approved in Canada, four were unable to receive referrals for desired surgical care domestically, and 11 sought procedures where expertise was lacking domestically.

**Table 2:** Participant Overview

**Characteristic**	**Count**
Recruitment Method for Study Participation	Word of mouth (n = 9); Facilitator referral (n = 8); Study advertising (n = 7); Media reports (n = 5); Online testimonials (n = 3)
Province or Territory of Residence	British Columbia (n = 18); Newfoundland & Labrador (n = 3); Ontario (n = 3); Quebec (n = 2); Alberta (n = 2); Nova Scotia (n = 2); Manitoba (n = 1); Northwest Territories (n = 1)
Procedure Sought Abroad	Orthopaedic surgery (n = 15); CCSVI therapy (n = 4); Eye surgery (n = 4); Bariatric surgery (n = 3); Cosmetic surgery (n = 3); Gastrointestinal surgery (n = 2); Dental surgery (n = 1)
Participant Ages	Average of 53 years; Median of 50 years; Range from 22 to 80
Sex	19 females; 13 males

The processes by which participants discovered, researched, and ultimately decided on pursuing medical care outside of Canada was extensively probed over the course of the interviews. Three distinct themes emerged from the accounts of the decision-making process: (1) information sources consulted during the decision-making process, (2) motivations, considerations, and timing regarding accessing medical care abroad, and (3) personal and professional supports drawn upon during the decision-making process. These themes are expanded on in the remainder of this section. As much as possible we have included verbatim quotations from the interviews in order to enable the participants themselves to ‘speak’ to these issues. Quotations were selected by the lead author as being a cogent representation of an issue assigned to a particular theme, and independently confirmed as such by the other authors.

### Information sources consulted

Participants identified four means of initially learning of medical tourism, namely: word-of-mouth (n = 13), non-targeted Internet searches (n = 10), print and televised media stories and advertising (n = 6), and familiarity with other countries’ health systems due to their having emigrated from them (n = 2). One person could not remember how they originally learned of medical tourism. For those who first learned of medical tourism online, the possibility of accessing care abroad usually emerged as an extension of researching treatments or trying to find an alternative means of accessing a surgery for which they were wait-listed domestically. “*I was looking for a magic bullet on the Internet…to address the…wait list issue that I was facing and so I had no idea what was out there…so I wasn’t actively seeking…I didn’t even know what I was looking for…I just thought there had to be something else…*” For those who learned about medical tourism from other people, former medical tourists (ranging in intimacy from close friends to one-off informal encounters), and friends and family with a passing knowledge of medical tourism served as important prompters. One exceptional case emerged where a Canadian family physician raised orthopedic care abroad with multiple participants amongst our dataset. While advertisements by medical tourism facilitators initiated some participants’ decision-making processes, news stories were more influential in raising participants’ awareness. Finally, for the two participants who were motivated by existing familiarity with non-domestic health systems, medical care outside of Canada was always seen as a possibility and there was no process of ‘discovering’ the option of care abroad.

Upon first learning of medical tourism, the vast majority of participants relied upon the Internet for detailed information. For these participants, it was used as a research tool to access the websites of facilitators, destination hospitals, joint replacement manufacturers, and empirical research. The Internet was also a powerful social tool, facilitating contact between participants and former medical tourists who provided personal anecdotes and advice. This communication sometimes took place in the context of online forums, though it was also common for participants to contact former medical tourists directly by e-mail. This sometimes resulted in having extended telephone conversations about their experiences. Participants also used the Internet to contact surgeons abroad directly for phone or e-mail consultations. These consultations were often informed by the sharing of diagnostic scans or reports between the prospective medical tourist and surgeon, the transmission of which was also facilitated by the Internet. The most common fact-finding approach amongst the medical tourists interviewed is characterized by this participant’s comments: “*I had had so little care here [in Canada] I figured it couldn’t be any worse over there. Maybe it could, I knew it was a third world country, but after I researched the hospital on the Internet and I talked to the four or five different people who went over there I had no concerns whatsoever*.” Six participants did not use the Internet at all in informing their decisions to go abroad, relying instead on family members in the destination country to obtain and relay information directly from and to the facility, the advice of former medical tourists, and/or the information provided by facilitation companies.

Although it was not directly probed, few participants discussed how they assessed the reliability of the information sources they consulted in the process of learning about medical tourism. Some outlined basic quality and reliability assessment practices. For example, one participant relied heavily on online physician rating sites, saying “*he [the surgeon] didn't have like any…bad write-ups online or anything… and when I didn’t see anything bad I figured well it must be okay, because I’ve looked up some doctors here for other things and I have seen bad comments.*” Another participant primarily relied upon a trusted hospital brand, saying “*But there’s no real deep research, it’s just uh a matter… [of] calling up your Mayo Clinic…on the computer screen, reading a bit and making a few calls and going from there.*” Other participants characterized themselves as savvy researchers, suggesting their skills extend to assessing the reliability of information, saying “*…now when some people…say oh they do online research…well sometimes they just mean that they’ve looked at a lot of ads, at advertisements for this kind of thing [procedure]…I didn’t do that, I looked for statistical surveys about the pros and cons of which procedure.*” While the majority of participants looked to the Internet as their primary information source, they commonly neglected to clearly delineate or discern what kind of information was ultimately accessed or who hosted it until they were prompted, while a minority made concerted attempts to convey the effort invested in seeking out what they thought was accurate and neutral information provided by third parties without commercial interests.

### Motivations, considerations, and timing

Participants identified many different motivations that spurred their initial consideration of medical tourism, which are summarized in Table 3. Despite this variety, all of the motivations discussed fall into the three broad categories of seeking procedures that are unavailable, wait-listed, or more costly in Canada. Cost was the primary motivation to pursue care abroad for four participants, all of whom sought cosmetic or dental surgeries that were available domestically for private purchase but not covered under public Medicare. Of the 14 participants that identified wait-listing as a key element motivating their trip, only seven ultimately pursued surgeries abroad that were available (and for which they could be put on a wait-list) domestically. The other seven concerned with wait-listing sought surgeries unavailable in their home provinces or territories. These alternate procedures were often described as more technically sophisticated and desirable than the domestic equivalent. These considerations became a keystone in their decision to travel for care that combined with, and sometimes eclipsed, the initial issue of wait-listing. Fourteen participants were solely motivated by procedure availability, seeking procedures that were not available to them in their provincial health or territorial system at the time of their medical travel. Reasons for this unavailability included the procedure not being approved by safety regulators, the patient being ineligible for surgery due to age or the absence of a diagnosis, or the lack of domestic surgical expertise to perform the surgery

**Table 3:** Primary motivations to pursue surgery abroad reported by participants

**Primary motivation(s)**	**# of participants**
Availability (where the procedure is not available domestically)	14
Wait-listing (where the procedure is available domestically)	7
Combined wait-listing & availability (where being wait-listed prompted a search for alternative surgeries not available domestically)	7
Cost (where the procedure is not covered by public Medicare)	4

Participants were faced with choosing which destination facility to visit. For many, the key deciding factor was the reputation of a surgeon they had found online and/or through social networks. The quality of the surgeon was assessed by looking at where s/he had trained, experience with the surgery, and/or testimonials from former patients. The perceived skill of the surgeon regularly outweighed more practical concerns, such as the possibility of encountering language barriers: “*I went for the surgeon. That was…the fundamental reason for going there. It was, not in terms of you know, where it was, no. I went for the surgeon*.” A group who deviated from this tendency to prioritize a particular surgeon over other variables were those who went abroad for CCSVI therapy. The majority who sought this procedure went to whichever clinic could treat them the soonest, regardless of its location. Another group of participants who did not choose their destination based on the surgeon were those returning to their countries of origin for surgery. Related to this, some made decisions based on previous international travel or living experience. The amount of in-hospital recuperation time offered also influenced destination choice. Those who stressed the importance of this put a high premium on the attention they would receive post-operatively, choosing the facility that would offer the most lengthy and attentive recuperation period. Proximity of the destination to Canada was relevant for some, but was never an overriding concern. Finally, cost was influential to a varying degree. For some, the affordability of care in the Global South greatly influenced their decision-making, as they would have accessed the surgery in a more developed nation if they had the money. For example, “*India was the cheapest of all of the ones that I researched or the least expensive rather than cheapest. I didn’t mean to cheapen it. The least expensive option was Chennai and that was a big factor…if I’d been a millionaire…I would have gone to Britain.*” Other participants reported finances as being low on the decision-making hierarchy, characterizing it as unimportant when compared with the other factors mentioned here.

There was great variety in the length of time it took participants to come to the ultimate decision to travel abroad for medical care, ranging from ten years to one week. Excluding the outlier of ten years, the average time from the discovery of the possibility of going abroad for surgery to contacting a facilitator or destination hospital with the intent to book a trip was six months (median = 3 months). This six month period was commonly spent researching potential destinations, assessing risks, speaking with other medical tourists, undertaking multiple calls or e-mails to facilitators and/or destination clinics, and in some cases attending local information seminars arranged by facilities seeking international patients. The time between booking and surgery was much more compressed, ranging from under one week to six months, with the average being two months (median = 2 months). For those who were assigned this two month period, it was seen as a reasonable and desirable amount of time to get things in order prior to travel and surgery while also giving time to arrange travel visas, where necessary.

### Decision-making supports

Two groups of people were commonly reported to have played supportive roles during participants’ decision-making processes. First, participants greatly appreciated and heavily considered anecdotal accounts from former medical tourists. These accounts were overwhelmingly positive endorsements of particular surgeons, destination hospitals, and/or the practice of medical tourism itself. In fact, the majority of participants sought advice from other medical tourists, ranging from reading online testimonials to speaking directly with such individuals. The following account is characteristic of the potency of these supportive encounters: “*I talked to some [former medical tourists], one guy especially who has been there a year before me…and his experience actually made me really go for it and have no, no more doubts…*[because] *he said ‘it’s totally up to standard, to Western standards, and a lot of people are trained in the West,’ and he said the service was so good he would send his daughter there on her own.”* Several participants also reported being contacted ‘out of the blue’ by former medical tourists who had heard about their upcoming trip through acquaintances or the local news media and offered strong support and additional advice, further validating their decision to go abroad for care. Second, family members played key supporting roles in helping to research and interpret information. For at least four participants this support extended to assistance with financing their medical care abroad. Although an important source of support, the opinions of friends and family had little impact on the outcomes of the decision-making process, with many participants adamant that they would have pursued their medical tour with or without the approval of their family or friends. This conviction was largely hypothetical, though, as none reported being seriously challenged by anyone during their decision-making.

Although most participants reported visiting with either their family physician or treating specialist during the time in which they were considering booking surgery abroad, they rarely sought physicians’ advice during the decision-making process. Instead, they more commonly waited to hear their regular physicians’ opinions on their decisions after having made a booking. Interestingly, the perception that one’s family physician or treating specialist would be unsupportive was cited as justification for not informing them of the plan to go abroad for surgery prior to booking. For example, one participant who did not speak with their family doctor explained that her surgery was “*None of his business… and he would have been prejudiced and he was prejudiced in any case*.” One of the most common reasons participants consulted with their regular physicians prior to going abroad was to acquire medical records or diagnostic tests in order to relay this information to destination physicians. While these physicians commonly complied with participants’ requests for records and/or tests, their reaction to the decision to go abroad for surgery ranged from supportive and caring to dismissive and dissuading. Remaining neutral and offering neither support nor discouragement was most common. Two examples of demonstrating uncommonly supportive family physicians include one who brought up the possibility of pursuing surgery abroad to patients before they had considered it and another who provided their personal cell phone number for the patient to provide their overseas surgeon in the case of an emergency to try and ensure a high degree of informational continuity. In both examples these physicians served as a significant source of support and guidance during decision-making.

Participants reported drawing on two industry-based sources of support in the course of their decision-making, those of medical tourism facilitators and clinics abroad. Ten participants reported using a medical tourism facilitator to arrange for care abroad. Many of them solely relied upon the facilitator for information about their procedure, the facility, and the surgeon abroad. For example, when asked *“Did you hear about the hospital that you went to in Bangalore from anyone else or was it just solely through the recommendation of the facilitation company?”* a participant replied *“Yeah. That was through the company that sent us; we had no idea where we were going.”* In the course of their decision-making, participants regularly reported having direct contact with their surgeon abroad via e-mail, phone, or less commonly, at in-person information seminars. Participants took the opportunity to ask questions regarding the potential risks of surgery, probable outcomes, and their suitability for the procedure. These interactions were greatly valued, and strengthened the resolve of many to access care abroad. When asked what the main deciding factor was in seeking surgery abroad, one participant appealed to these interactions with the surgeon, saying: “*You know it probably was the doctor, I can’t think of anything else…I was in touch with him two or three times, he called me by telephone and spoke to him about a lot of the concerns and things and…I think…he was the main factor*."

## Discussion

The opportunity to seek care abroad through the medical tourism industry creates new means of acting on motivations and needs that have likely always underpinned surgical decision-making in domestic contexts but may have been constrained by structural arrangements. The current Canadian model of accessing surgical care privileges the position of the expert over the non-expert by requiring patients to seek referrals to tertiary care providers from their primary care physicians [40], with the exception of surgical care not provided through the public health care system. The medical tourists we spoke with, however, tended to seek advice and information from many sources other than their regular physicians or other members of the medical community and were ultimately responsible for deciding when and where care was to be delivered as long as they could find a willing surgeon abroad. As such, involvement in medical tourism changed participants’ typical enactment of the ‘patient role’ and the means by which they decided on medical treatment. The significance of these changes is discussed below in regard to participants’ layered motivations, the timelines of care commonly seen, and the sources of information accessed and relied upon in their decision-making processes.

The current literature on medical tourism broadly categorizes patient motivations, typically attributing a single motivator to medical tourists from any given health system [e.g., [36,41]. In these accounts, Canadian medical tourists have generally been afforded only one motivation for accessing medical care abroad, that of wait-lists [e.g., [42]. While wait-lists and wait-listing played a role in motivating many of the participants to look for care outside of Canada, it is important to note that participants provided examples of all three of the regularly cited motivations for medical tourism, those of procedure cost, availability, and wait-listing [12]. Given the lack of universal coverage by public insurance plans for dental and cosmetic surgeries in Canada, it was not surprising to have heard accounts of Canadians choosing to access these treatments at more affordable rates abroad. Cost also served as a secondary motivator for many, serving to promote more affordable destinations once participants were seriously researching their options for a specific procedure abroad. The role of procedure availability played a far more nebulous role as a motivator when compared with cost. It played a primary motivating role for those seeking experimental surgeries (e.g., CCSVI, eye surgery for retinosis pigmentoria), as there are no similar treatments available in Canada. It also served as a primary motivator for those who were unable to get specialist referrals domestically, or whose conditions were deemed inoperable by their domestic physician. Similarly, previous discussions of medical tourists have rarely accounted for individual backgrounds that might influence the countries they visit for medical care. Meanwhile, our analysis suggests that previous exposure to foreign countries, either through travel or emigration, might bear influence on the destinations they ultimately select.

For many participants who were *initially* motivated to explore the option of care abroad as a result of having been wait-listed or being worried about the prospect of one, the availability of procedures performed abroad which were perceived to be technically superior supplanted this initial motivation. This supplantation of availability with wait-listing was seen repeatedly for those who sought hip resurfacing, an alternative to a total hip replacement, and vertical sleeve gastrectomies, a form of gastric bypass surgery. Meanwhile, the desire to avoid a wait list for the same surgery available domestically played a role in only six participants’ accounts. These layered motivations suggest that the decision to access surgery abroad cannot be crudely reduced to a single motivator, and that contextual elements and secondary motivators should be considered alongside the most powerful motivator in any given account. Perhaps unsurprisingly, a common element to all of the accounts was a strong hope that the surgery sought abroad would improve the participants’ quality of life, as a sentiment of the importance of achieving good health at any cost emerged in many of the interviews. If the barrier to a good quality of life through surgery was perceived to be availability, cost, or a lengthy domestic wait list, participants were compelled to find the means abroad to overcome them, regardless of the procedure’s objectively scored urgency and/or necessity.

Notably, the particular contexts of individual destination countries were relatively unimportant in most of participants’ decision-making processes. More specifically, the particular details of a destination country’s wealth, politics, history, language, and other characteristics were of minor importance when compared to the reputation of the surgeon and the facility. In this way, the ‘global’ aspect of medical tourism is both effaced and affirmed as the differences between potential destination nations disappear and are replaced by placeless images of homogenous clinical spaces in the imaginations of medical tourists. This finding departs from some of the conceptual decision-making models that have been published in the tourism studies literature that have emphasized the importance of destination nation characteristics to potential medical tourists’ decision-making processes [e.g. [43,44].

While word-of-mouth information sharing has been noted as an important factor in other studies of surgical patient decision-making [e.g., [45], the degree to which word-of-mouth recommendations and endorsements serve as a primary consideration for medical tourists was found to be remarkably consistent. This factor was found to be equally important in a recent study of Omani medical tourists [46]. Another consistency among our participants was a general lack of consultation with their regular physicians during the decision-making process. Within the Canadian system, family doctors and other primary care physicians serve as a keystone in patients’ pursuit of the majority of elective surgical care by assessing need, providing counseling, and arranging for appointments with specialists who relay detailed information about the risks and benefits of surgery [47]. Despite these established roles in supporting patients’ medical decision-making, far more valued was the advice and support provided by other medical tourists. This mirrors Kangas’ [48] findings amongst Yemeni medical tourists, whose considerations of and where to go abroad for medical care were deeply informed by word-of-mouth networks recommending particular destination clinics and physicians.

While the value placed on the expertise from former medical tourists by those engaging in decision-making around pursuing care abroad should not be discounted given their first-hand knowledge of what to expect from particular hospitals or surgeons, the conspicuous absence of a neutral, yet informed, third party informing the decision-making process must be noted. Positive testimonials have been found to skew the interpretation of surgical risk, resulting in a disproportionate weighting of the potential positive outcomes even when presented with the statistical likelihood of the potential negative outcomes [31]. This raises concern about whether or not medical tourist are always in a position to give informed consent to care abroad based on the information they have considered, given that such consent requires a sound understanding prior to surgery of their condition, success rates, treatment options, and risk of complication [30,48]. Given the current lack of comprehensive and neutral guidance available to medical tourists, there have been a number of calls for stronger informational support by third-parties that do not have a vested financial interest in medical tourism [33,49,50]. Knowing that former medical tourists play such an influential role in informing prospective medical tourists could be useful to those designing such interventions, wherein former medical tourists could be targeted in informational campaigns with the intent of having them pass such information along to those contacting them for advice. Furthermore, awareness of our finding of the wide variance in the timing of the pre-booking research period by medical tourists and the relative two month consistency of the post-booking period could aid in developing strategies to disseminate informational interventions that are sensitive to the timeline of prospective medical tourists’ informational needs.

### Wider relevance

The growth of the medical tourism industry has clear implications for global health equity [3,51]. By extension, the decision-making considerations of individual medical tourists and the information they access is tied to the development of this industry and its potential to operate equitably and ethically. One commonly cited health equity concern pertains to the use of public resources by the private medical tourism industry [3]. Although much consideration has been given in the medical tourism literature to the potential for patients to require expensive follow-up care in their home countries [12,52,53], our findings show that most of the medical tourists we spoke with sought out some degree of advice or logistical support from their family physicians and treating specialists *prior* to going abroad (but not necessarily before booking the procedure). As primary care consults and many lab costs in Canada are covered by public funding, this is another potential pathway through which public funds support the operation of this private, for-profit industry. More research attention needs to be given to uncovering the ways in which patients’ home health care systems indirectly support the medical tourism industry in order to inform health equity debates surrounding this global health services practice.

In terms of health equity in medical tourism destination countries, it is thought that medical tourists traveling to economically developing nations may exacerbate existing health inequities by raising the cost of care and/or lessening the availability of specialists to local citizens through increasing demand for their services [54]. Meanwhile, it has also been suggested that the revenues from medical tourists could be used to cross-subsidize the care local patients in order to mitigate potential negative health equity impacts [11]. Should the appropriate redistributive financing mechanisms and regulations be developed in destination countries or at individual facilities, medical tourists’ willingness to incur added fees to access more equitable care is likely contingent on their understanding of the health challenges faced by economically developing destination nations. Our findings suggest a general lack of awareness amongst the medical tourists we spoke with in terms of their knowledge of contextual details of the particular destinations they chose to travel to during decision-making about seeking care abroad. In fact, consideration of the destination country in any way held little weight in the decision-making process. Medical tourists may more carefully consider health equity in the destination and the impacts of their decisions if prompted to do so in informational interventions or through other means and mediums.

While this analysis has focused specifically on Canadian medical tourists, our findings have relevance for medical tourists from other nations. Here we highlight three such issues. First, while the contextual details of medical tourists’ home health systems may differ, they seek care in a common global marketplace. Our findings have confirmed that this marketplace is largely mediated through the Internet, where much of the information that prospective medical tourists consider is accessed online. Second, amongst our participants, context-specific domestic health system considerations informed their decision making processes. For example, particular strengths (e.g., universal access) and weaknesses (e.g., care rationing) of the Canadian public health care system underlay the kinds of surgeries that were sought out-of-country and the motivations to go abroad. Patients exiting other countries with universal public healthcare coverage, such as the United Kingdom and Norway, may too be motivated to go abroad for the same reasons at the Canadian medical tourists we spoke with. Third, upon entering the same global marketplace, potential medical tourists are exposed to many of the same web pages and advertisements regardless of the regulatory, legal, and political environments from which they will depart. This reality underscores the importance of thinking of this patient group as influenced, but not strictly defined by, their home health system contexts.

### Limitations

As recruitment was limited to English, we have excluded French-language participants as well as other linguistic minorities who do not have spoken English fluency. Additionally, given the difficulty of recruiting the study population and our subsequent reliance on snowball sampling, there is likely a disproportionate focus on particular surgeries sought in specific destinations and medical tourists from certain regions of Canada. Finally, our reliance on the retrospective recollections may have resulted in the omission of key details and/or heightened the bias of their recall of events when compared with a prospective approach to data collection.

## Conclusions

In this article we have presented the findings of interviews with 32 Canadian medical tourists, with a specific focus on their decision-making processes regarding seeking surgery abroad. Our analysis confirms accounts of medical tourism that attribute its growth to the ability of the Internet to connect distant parties with mutual interests to one another [55,56]. That prospective Canadian medical tourists relied upon the Internet to put them in touch with information about clinics, surgeons, and other medical tourists is therefore not surprising. What is noteworthy, however, is the degree to which the opinions and advice of other medical tourists informed participants’ awareness of medical tourism and their ultimate decision to travel abroad for care. This adds evidence to existing concerns that prospective medical tourists may have limited access to accurate and unbiased sources of information about their treatments, especially online [16,33]. The creation of such sources could greatly benefit all medical tourists considering surgery abroad by providing a more complete picture to inform their decision-making.

The accounts provided by Canadian medical tourists complicate existing broad characterizations in the medical tourism literature that attribute the motivations of medical tourists leaving any given country to a single motivating force, such as cost of care, wait-listing, or the availability of procedures. The medical tourists we interviewed made it clear that all three of these motivators were at play in their decision to seek care abroad, often in combination with one another. Future accounts or investigations of medical tourism would benefit from a more nuanced consideration of the layered motivations that are driving patients to seek medical care abroad, rather than accepting the current broad-stroke accounts that attribute a single motivator to the medical tourists of any one locale. It is also important that future research addresses the quantitative knowledge gaps rife in medical tourism research to provide broader context and grounding for the trends described in this analysis and other qualitative studies.

## Abbreviations

CCSVI: Cerebro-Spinal Venous Insufficiency; US: United States.

## Competing interests

The authors declare they have no competing interests.

## Authors' contributions

RJ undertook data collection, coding, wrote the first draft, and undertook the bulk of subsequent edits. VAC developed the study protocol, contributed to coding scheme development, was involved in interpreting the findings, and closely edited multiple drafts. JS contributed to the data collection instrument and coding scheme, confirmed the RJ’s interpretation of the findings, and provided feedback on drafts. All authors reviewed and approved of the submitted manuscript.
